# Supplementary material for: The interactome of intact mitochondria by cross-linking mass spectrometry provides evidence for coexisting respiratory supercomplexes
Source: Mol Cell Proteomics. 2017 Dec 8;17(2):216–32. doi: 10.1074/mcp.RA117.000470 (PMC5795388; doi:10.1074/mcp.RA117.000470)
Supplement: Supplemental Data [file supp_RA117.000470_133922_0_supp_23978_fzffwf.zip › spectra_annotation/mito_DR_spectra_annotation/483-1-5-1-21-1.pdf]

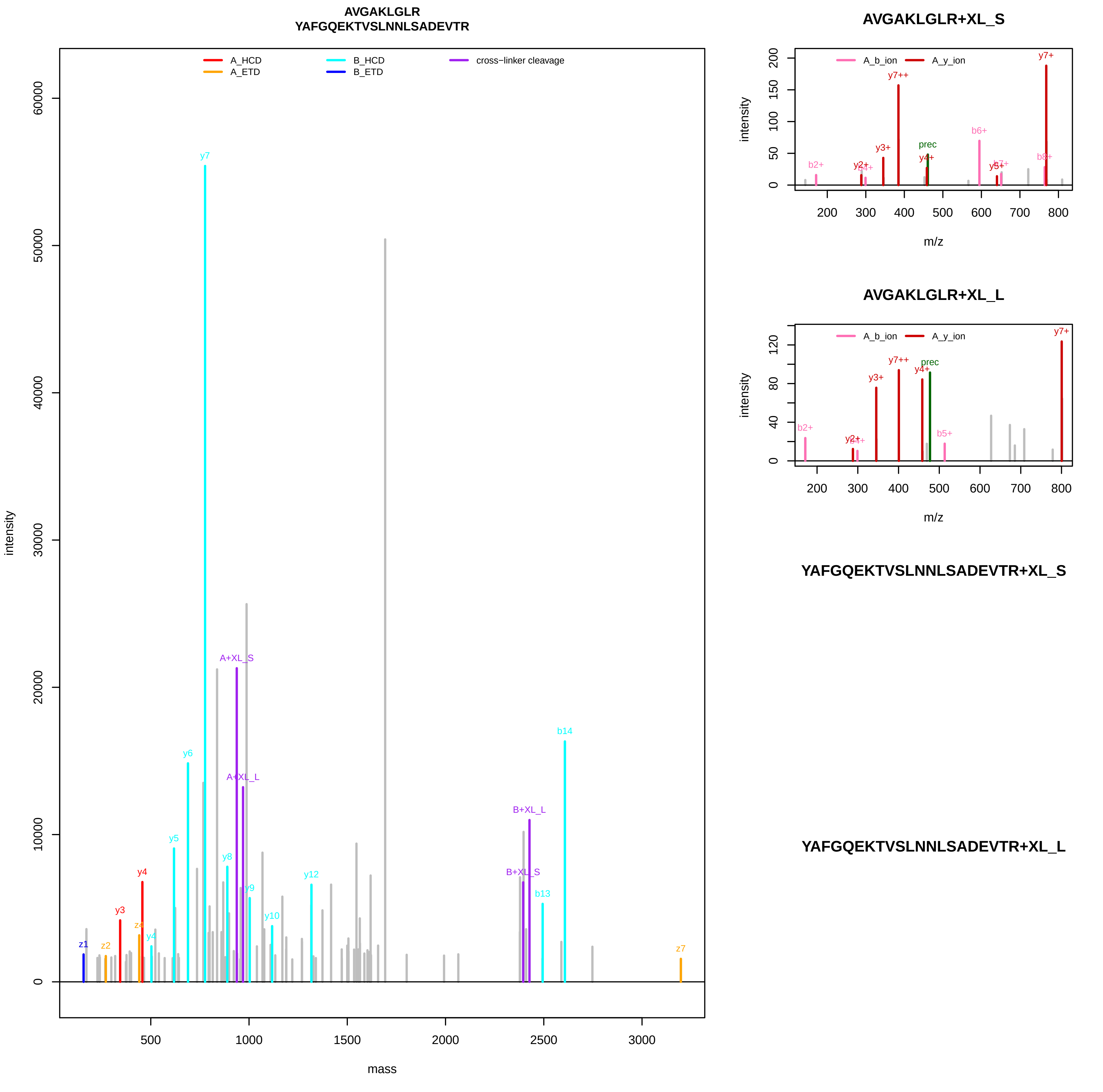
AVGAKLGLR
YAFGQEKTVSLNNLSADEVTR
A_HCD
A_ETD
B_HCD
B_ETD
cross−linker cleavage
intensity
mass
y7
A+XL_S
A+XL_L
y6
y5
y8
y9
y10
y12
b14
b13
B+XL_L
B+XL_S
y4
y3
z1
z2
z4
z7
AVGAKLGLR+XL_S
A_b_ion
A_y_ion
m/z
prec
AVGAKLGLR+XL_L
YAFGQEKTVSLNNLSADEVTR+XL_S
YAFGQEKTVSLNNLSADEVTR+XL_L
